# Polyphenols from Cocoa and Vascular Health—A Critical Review

**DOI:** 10.3390/ijms10104290

**Published:** 2009-11-20

**Authors:** Gerald Rimbach, Mona Melchin, Jennifer Moehring, Anika E. Wagner

**Affiliations:** Institute of Human Nutrition and Food Science, Christian Albrechts University 24118 Kiel, Germany; E-Mails: melchin@foodsci.uni-kiel.de (M.M.); moehring@foodsci.uni-kiel.de (J.M.); wagner@foodsci.uni-kiel.de (A.E.W.)

**Keywords:** cocoa, chocolate, polyphenols, cardiovascular disease, endothelial function

## Abstract

Cocoa is a rich source of dietary polyphenols. *In vitro* as well as cell culture data indicate that cocoa polyphenols may exhibit antioxidant and anti-inflammatory, as well as anti-atherogenic activity. Several molecular targets (e.g., nuclear factor kappa B, endothelial nitric oxide synthase, angiotensin converting enzyme) have been recently identified which may partly explain potential beneficial cardiovascular effects of cocoa polyphenols. However cocoa polyphenol concentrations, as used in many cell culture studies, are not physiologically achievable. Bioavailability studies indicate that plasma concentrations of cocoa polyphenols following dietary intake are low and in the nanomolar range. Human studies regarding the effect of cocoa polyphenols on vascular health are often underpowered and lack a rigorous study design. If dietary cocoa polyphenol intake is due to chocolate its high energy content needs to be taken into account. In order to determine potential health benefits of cocoa polyphenols large scale, long term, randomized, placebo controlled studies, (ideally with a cross-over design) as well as prospective studies are warranted.

## 1. Introduction

There is experimental evidence suggesting that cocoa polyphenols may mediate beneficial effects on vascular health [1–3]. The present review critically evaluates studies on cocoa polyphenols and vascular health as conducted in cultured cells, laboratory rodents and humans over the last decade. First bioavailability studies with cocoa polyphenols are summarized. Secondly potential molecular targets of cocoa polyphenols are discussed. Thirdly an overview of the human studies on cocoa polyphenols and cardiovascular disease prevention is given. Finally merits and limitations of these studies are discussed and future challenges in cocoa polyphenol research are presented.

## 2. Bioavailability of Polyphenols in Cocoa

**Figure 1.**
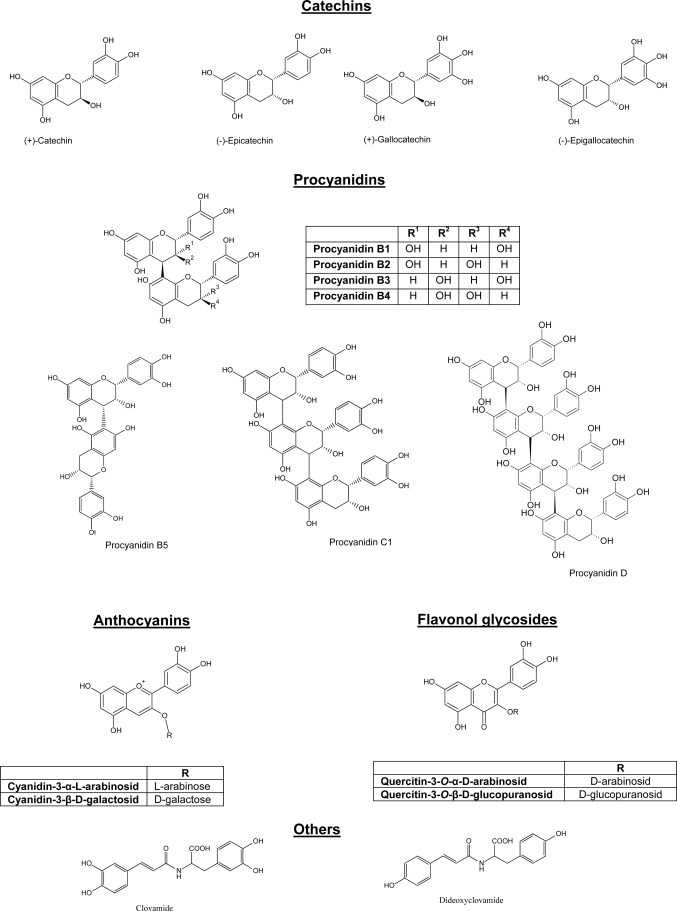
Chemical structure of cocoa polyphenols.

Cocoa beans (*Theobroma cacao*) have been used for a long period of time as major ingredient of cocoa and chocolate [4,5]. Cocoa is a rich source of polyphenols. Cocoa beans contain approximately 6–8% polyphenols by dry weight [6]. Polyphenols identified in cocoa beans and cocoa products comprise mainly catechins, flavonol glycosides, anthocyanins and procyanidins. As far as procyanidins are concerned, up to decamer ones have been identified in cocoa [7]. The chemical structure of selected cocoa polyphenols is given in Figure 1.

The biological activity of cocoa polyphenols largely depend on their bioavailability [8]. The bioavailability of cocoa polyphenols has been measured in several studies in humans (Table 1). Polyphenols were given as cocoa rich beverages or chocolate. Monomeric flavonoids as well as dimeric and trimeric procyanidins have been detected in human plasma after consumption. The peak plasma concentration of flavonols was determined 2–3 hours after ingestion [9,10]. Plasma concentrations of cocoa polyphenols were often in the nanomolar or low micromolar range. Donovan and coworkers demonstrated that commercial chocolate samples available around the world contain a predominance of the less bioavailable (−)-catechin enantiomer as compared to the (+)-catechin which is present in most other plant derived foods. This may explain the relatively low bioavailability of catechins from chocolate and cocoa-containing products [11]. The food matrix seems to be an important factor that may affect the bioavailability of cocoa polyphenols. Cocoa powder dissolved in milk, as one of the most common ways of cocoa powder consumption, did not change the bioavailability of cocoa powder flavonoids in healthy humans [12]. Furthermore, lipid and protein rich meals did not affect the bioavailability of cocoa polyphenols. However the uptake of flavonols in humans could be increased significantly by concurrent consumption of carbohydrates [13].

It is unclear if and to what extend higher oligomeric procyanidins from cocoa are absorbed. However, the biological activity of higher procyanidins may be at least partly attributed to their colonic breakdown products, including phenolic acids [14]. Data regarding the tissue distribution of cocoa polyphenols in laboratory rodents are currently missing.

Within this review we mainly focus on potential cardiovascular health benefits of cocoa polyphenols. However it needs to be taken into account that cocoa beans contain also lipids, sterols, minerals and trace elements which may also affect vascular health as reviewed by Steinberg *et al*. [15].

## 3. Putative Health Benefits of Polyphenols from Cocoa and Chocolate

### 3.1. Studies in Vitro and in Cultured Cells

*In vitro* studies, as well as studies in cultured cells, have identified several cellular and molecular targets by which cocoa polyphenols may mediate beneficial cardiovascular effects (Table 2). In fact it has been shown that cocoa polyphenols may prevent and/or inhibit the oxidation of LDL which is a key event in atherogenesis [26–30]. Furthermore cocoa polyphenols inhibit lipoxygenase activity *in vitro* [31]. However, comparing the 5-lipoxygenase-inhibitory effect of epicatechin (present in cocoa) with those of epigallocatechin-gallate (present in green tea) it was found that the latter flavonoid was at least one order of magnitude more potent [32]. There is experimental evidence from studies with rat liver microsomes that cocoa polyphenols may decrease NADPH-dependent lipid peroxidation and linoleic acid autoxidation [33].

Further studies in cultured cells suggest that cocoa polyphenols may exhibit anti-inflammatory activity by down regulating the production of pro-inflammatory cytokines including IL1β, IL2, IL4, IL6 and TNF-α [34–38]. Activated macrophages can generate large amounts of nitric oxide from l-arginine by the action of inducible NO Synthase (iNOS). Overproduction of NO by macrophages has been associated with chronic inflammation [39]. The transcription factor NF-κB, in cooperation with AP-1, cooridnates the expression of iNOS and pro-inflammatory cytokines [40]. Cocoa polyphenols may decrease inducible nitric oxide production by inhibiting iNOS gene expression due to NFκB and AP1 dependent signal transduction pathways [41,42]. Contrary other studies in cultured RAW264.7 murine macrophages indicate that oligomeric procyanidins such as procyanidin C2, as present in cocoa, exhibit pro-inflammatory activity [43]. Mao *et al*. have shown that the smaller fraction of cocoa polyphenols (monomer-tetramer) consistently decreased IL1β expression in blood mononuclear cells, while the larger oligomers (pentamer-decamer) increased its expression [36].

Studies in endothelial cells suggest that cocoa polyphenols such as epicatechin inhibit arginase-2 mRNA expression and activity levels which in turn may result in a higher availability of the vaso-relaxing molecule nitric oxide in the vascular wall [44]. This finding is in line with studies using isolated rabbit aortic rings in which endothelium dependent relaxation and an increase in nitric oxide synthase has been observed. Karim and co-workers [45] studied the effect of polymeric procyanidins on endothelium dependent relaxation (EDR) in aortic rings from New Zealand White rabbits. Polymeric procyanidins (tetramers through decamers of catechins) caused EDR in aortic rings. Furthermore polymeric procyanidins significantly increased Ca^2+^dependent NOS activity whereas monomers, dimers and trimers of catechins exhibited no such activity.

Cocoa procyanidins inhibit metalloproteinase-2 expression and activation in smooth muscle cells which may contribute to the anti-atherosclerotic effects of cocoa [46].

Interestingly cocoa polyphenols have also been shown to inhibit ACE activity [47] which may result in a decrease of blood pressure. ACE is centrally involved in the regulation of the rennin-angiotensin system. Angiotensin causes blood vessels to constrict resulting in increased blood pressure. Thus ACE inhibition is a therapeutic approach in blood pressure regulation. As observed for the purified ACE enzyme, ACE activity in kidney membrane was inhibited by 100 μmol/L of dimer and hexamer epicatechin [47] a concentration which is not physiologically achievable.

Furthermore cocoa polyphenols circulate as conjugated metabolites whereas in most *in vitro* studies and studies in cultured cells non-conjugated cocoa polyphenols have been used. In this context cell culture studies using conjugated cocoa polyphenols are warranted. Furthermore the underlying mechanisms involved into the cellular uptake of cocoa polyphenols as well as its cellular concentrations and subcellular distribution need to be established.

### 3.2. Studies in Laboratory Animals

*In vitro* studies and studies in cultured cells are partly supported by studies in laboratory animals indicating that cocoa polyphenols may decrease LDL-oxidation, as well as other biomarkers of lipid peroxidation [4]. Furthermore studies in hypercholesterolemic rabbits [48] suggest that cocoa polyphenols may prolong LDL-oxidation lag time and decrease area of atherosclerotic lesions in the aorta (Table 4). Studies in hamsters report an increase in HDL as well as a decrease in LDL and triglyceride levels [49]. Furthermore cocoa procyanidins significantly reduced plasma cholesterol and increased steroid excretion in rats fed a high cholesterol diet [50]. In obese diabetic mice and rats a decrease in blood glucose as well as a decrease in 8-isoprostane levels has been observed [51]. However in other studies in rats no effect of cocoa polyphenols on biomarkers of lipid peroxidation in liver and heart has been reported [52].

The inhibition of arginase activity has been reported to improve endothelium vasodilating relaxation. In this context Schnorr *et al.* have found that cocoa flavonols lower vascular arginase activity in rat kidney. Arginase competes with endothelial nitric oxide synthase for l-arginine as the substrate. Thus an inhibition of arginase activity can be associated with elevated endothelial NO levels [44]. Potential mechanisms by which cocoa polyphenols may affect vascular health are summarized in Table 3.

### 3.3. Studies in Humans

There is evidence suggesting that cocoa polyphenols may also positively affect vascular health in humans. Several studies in humans suggest that cocoa polyphenols decrease LDL-oxidation [19,57,58]. Furthermore, an improvement of plasma antioxidative status due to cocoa polyphenols has been shown [9]. Some studies indicate an increase in plasma HDL-cholesterol [19,59,60], a decrease in plasma triglyceride and a decrease of biomarkers of lipid peroxidation such as TBARS [17,61] and F_2_-Isoprostanes [62] following cocoa polyphenol consumption.

Importantly dietary cocoa polyphenols improve endothelial function by increasing vascular-NO-synthase activity [63–67]. This in turn may lead to a decrease in systolic and diastolic blood pressure [68–72]. The magnitude of blood pressure reduction due to cocoa polyphenols is often relatively low [73]. Blood pressure lowering effects were more evident in hypertensive than in normotensive volunteers [71,73]. However other studies did not report a beneficial effect of cocoa polyphenols on blood pressure, and flow mediated dilatation [63]. Balzer *et al*. [74] studied the effect of flavonol-containing cocoa on vascular function in medicated diabetic patients in a double-masked, randomized, controlled trial. The key observation was an acute effect and a later chronic effect of cocoa flavonols on endothelial function as evidenced by improvements in FMD. However, over a 6-week period, flavonol-rich cocoa did not modify vascular function in subjects with coronary artery disease. Furthermore no differences in soluble cellular adhesion molecule-concentration in plasma and forearm blood flow response to ischemia were evident [75].

Several studies report anti-aggregatory effects of cocoa polyphenols [25,61]. However the effects of cocoa polyphenols on platelet aggregation are rather modest and possibly large amounts need to be ingested to exhibit a similar anti-aggregatory effect as reported for aspirin [76]. Nevertheless in a recent study by Flammer and coworkers dark chocolate induced coronary vasodilation, improved coronary vascular function and decreased platelet adhesion 2 h after consumption of 40 g of dark chocolate containing 70% cocoa. These immediate effects were accompanied by a significant increase in plasma epicatechin levels [77].

Although there is experimental evidence that cocoa polyphenols may act as free radical scavengers *in vitro*, it is unlikely that cocoa polyphenols exhibit significant free radical scavenging activity *in vivo*. Cocoa polyphenols are present at low concentrations in the human plasma and mostly in the conjugated form [18]. Conjugation of flavonoids with glucoronic acid and sulphate blocks radical scavenging hydroxyl groups [78,79]. Furthermore the free radical scavenging activity of human plasma is mainly attributable to vitamin C, vitamin E, bilirubin and urate which occur in many times higher concentration as compared to cocoa polyphenols [80].

Hypertension is a leading risk of death in the world [87]. If a patient is diagnosed with high blood pressure anti-hypertensive drugs need to be prescribed. Functional food and their nutraceuticals may have a preventive potential, but they do not play a therapeutic role if the disease has already occurred. Although high flavonol cocoa polyphenols improved endothelial function, it did not improve the effects of exercise on body fat in obese adults [88]. From a nutritional point of view it is questionable whether one should increase the intake of dietary polyphenols due to cocoa-rich products such as chocolate, which is rich in fat and sugar and thereby high in energy. In the study by Taubert *et al*. consecutive daily doses of 100 g dark chocolate over two weeks increased the caloric intake by 480 kcal per day. Although systolic blood pressure was decreased by 5 mm Hg, in the long run these extra calories would probably lead to an increase in body weight and may counteract potential beneficial effects on vascular health [89]. Thus products low in sugar and fat should be preferred.

It needs to be taken into account that many human studies on cocoa polyphenols and vascular health as reported in the literature, were not placebo-controlled. Furthermore most studies regarding potential health benefits of cocoa polyphenols did include only small number of volunteers as summarized in Table 5. In fact many human intervention trials with cocoa polyphenols seem to be underpowered. Another problem with these studies is that rigorous study design is missing and compliance of the volunteers as well as plasma polyphenol concentrations have often not been reported. In order to determine health benefits of cocoa polyphenols large scale, long term randomised placebo-controlled studies (ideally with a cross-over design) are warranted [90]. Furthermore prospective studies on cocoa polyphenols need to be conducted in the future.

## 4. Conclusions

There is epidemiological evidence suggesting that consumption of fruits and vegetables appear to have a protective effect against coronary heart disease [91]. Furthermore, the intake of certain flavonoid rich food items may be inversely related to coronary heart disease [92,93]. However, only few epidemiological data are currently available for cocoa polyphenols. In a cohort of elderly men, cocoa intake was inversely associated with blood pressure and 15-year cardiovascular and all-cause mortality [94]. It is suggested that before drawing conclusions, confirmation by further observational and experimental studies on cocoa polyphenols is needed. Mink *et al*. [95] studied flavonoid intake and cardiovascular disease mortality in a prospective study in postmenopausal women. Individual foods associated with a reduction of cardiovascular disease risk included bran, apples and pears, red wine, grapefruit, strawberries and chocolate.

Hooper and coworkers have recently conducted a meta-analysis on flavonoids, flavonoid-rich foods and cardiovascular disease risk. One hundred thirty-three trials were included into this meta-analysis. Importantly chocolate increased FMD and reduced both systolic and diastolic blood pressure [96]. Janszky *et al*. [97] assessed the long-term effects of chocolate consumption amongst patients with established coronary heart disease in a population based cohort study comprising >1100 non diabetic patients. Chocolate consumption was associated with a significantly reduced cardiac mortality in patients surviving the first acute myocardial infarction.

In a recent review it has been stated that most cocoa polyphenol studies in humans over the last decade have been mainly funded by industrial sponsors. Thus there may be potential for research bias [98]. Nevertheless several studies consistently reported beneficial effects of dietary polyphenols from cocoa on vascular health. However, it should be considered that the products used in controlled studies often contain much higher polyphenol contents than most of the commercially available products [94,99]. Since flavonols exhibit a bitter taste manufacturers have established processing techniques for cocoa which eliminate the bitterness together with the flavonoides [100]. As much as 90% of the flavonoids may be lost due cocoa processing [76].Thus, it needs to be established whether the consumption of products with a lower polyphenol content are associated with any health benefits in humans. Furthermore the food industry is encouraged to label the flavonoid content on their cocoa derived products.

## Figures and Tables

**Table 1.** Human bioavailability studies on cocoa polyphenols.

**No. Subjects**	**Age range (mean)**	**BMI (kg/m^2^)**	**Dietary source of polyphenols**	**Polyphenol content**	**Plasma concentration**	**Reference**
8	25–55 (40 ± 15) (± SD)	23.94 ± 2.35 (± SD)	Dark chocolate: 40 g 80 g	Epicatechin: 82 mg 164 mg	Epicatechin: 0.383 μmol/L (t = 2 h) 0.7 μmol/L (t = 2.57 h)	[16]
20	20–56	23.8 ± 0.79 (± SEM)	Semi-sweet chocolate baking bits: 27 g 53 g 80 g	Total procyanidins (epicatechin): 186 (46) mg 365 (90) mg 551 (136) mg	Epicatechin (t = 2 h): 0.133 μmol/L 0.258 μmol/L 0.355 μmol/L	[17]
13	26–49	23.2 ± 1.2 (± SEM)	105 g semi-sweet chocolate baking bits (of which 80 g procyanidin-rich chocolate)	557 mg total procyanidins (137 mg epicatechin)	0.257 μmol/L epicatechin (t = 2 h)	[10]
5	30–33 (31 ± 1) (± SD)	22.5 ± 1.3 (± SD)	96 g chocolate 66 g cocoa	Total polyphenols (epicatechin): 2.74 g (760 μmoL) 2.73 g (760 μmoL)	Total epicatechin (t = 2 h): 4.77 μmol/L 4.92 μmol/L	[18]
23	21–62	–	22 g cocoa powder + 16 g dark chocolate	466 mg total procyanidins (111 mg monomers)	0.036 nmol/L epicatechin (t = 2 h)	[19]
11	20–55 (39 ± 5) (± SD)	24 ± 3 (± SD)	37 g high- vs. low-procyanidin chocolate	Total procyanidins: 4 mg/g vs. 0.9 mg/g	0.212 μmol/L vs. 0.011 μmol/L epicatechin (t = 2 h)	[20]
16	22–49	–	300 mL cocoa beverage (18.75 g flavanol-rich cocoa powder)	897 mg total epicatechin & procyanidins	1.043 μmol/L epicatechin (t=2 h)	[21]
5	23–34	–	Cocoa beverage (0.375 g cocoa/kg bw)	*Per g cocoa*: 12.2 mg monomers, 9.7 mg dimers, 28.2 mg procyanidins	0.041 μmol/L dimer B2, 5.92 μmol/L epicatechin, 0.16 μmol/L catechin (t = 2 h)	[22]
6	23–39	23.1 ± 0.7 (± SEM)	400 mL flavanol-rich cocoa beverage (37.5 g cocoa); 2 days	*Per g cocoa*: 12.2 mg monomers, 9.7 mg dimers, 20.2 mg procyanidins	0.08 μmol/L dimer B2, 4.11 μmol/L epicatechin, 0.4 μmol/L catechin (t = 2 h)	[23]
18	–	–	25 g semi-sweet chocolate chips	220 mg flavanols & procyanidins	0.427 μmol/L epicatechin (t = 2 h)	[24]
32	31–49 (40 ± 9) (± SD)	26 ± 4 (± SD)	Cocoa flavanol & procyanidin supplementation for 28 days	234 mg/d flavanols & procyanidins	0.116 μmol/L epicatechin 0.091 nmol/L catechin (t = 28 d)	[25]

**Table 2.** *In vitro* and cell-culture studies performed with cocoa polyphenols.

**Cell type**	**Treatment**	**Outcomes**	**Reference**
Human LDL	Cocoa powder extract (5 μmol/L GAE°) vs. pure catechin (5 μmol/L)	LDL oxidation ↓	[26]
Liposomes & human LDL	Cocoa catechin monomers & procyanidin fractions (0.1–10.0 μg/mL)	LDL oxidation ↓	[27]
LDL	220 mL cocoa drink (cocoa concentration: 1.5, 2.0, 2.5, 3.0, 3.5%)	LDL oxidation ↓ dose-dependent	[28]
LDL	Catechin, epicatechin, procyanidin B2, procyanidin C1, cinnamtannin A2 (0.125, 0.25, 0.5, 1.0, 2.0 μg/mL)	LDL oxidizability ↓	[29]
Human LDL & VLDL	Dark chocolate, cocoa, milk chocolate, hot cocoa mixes (126, 224, 52.2, 8.2 μmol/g total phenols)	Lag time of LDL & VLDL oxidation ↑	[30]
LDL & VLDL	Dark chocolate & cocoa powder (containing fat or defatted) vs. cocoa butter	LDL & VLDL oxidizability ↓	[49]
Rat liver microsomes	Cacao liquor	NADPH-dependent lipid peroxidation ↓ Linoleic acid autoxidation ↓	[33]
Recombinant human 5-LOX	Cocoa epicatechin & procyanidins (10 μmol/L)	5-LOX activity ↓ Proinflammatory mediators (LTB4, LTC4, LTD4) ↓	[32]
Isolated rabbit 15-LOX-1 Recombinant human platelet 12-LOX	Cocoa procyanidins (monomers through decamers; 2.9 mg/mL) Epicatechin & procyanidin decamer	15-LOX-1 activity ↓ dose-dependent 12-LOX activity ↓ dose-dependent	[31]

Human PBMC *	Cocoa procyanidins (monomers through decamers; 25 μg/mL)	IL-1β secretion ↑ IL-2 expression ↓ IL-4 expression & secretion ↓	[34]
Human PBMC *	Cocoa procyanidins (monomers through decamers; 25 μg/mL)	IL-1β transcription & secretion ↑ (pentamers-decamers) IL-1β transcription & secretion ↓ (monomers-tetramers)	[36]
Human PBMC *	Cocoa procyanidins (monomers through decamers; 25 μg/mL)	Secretory IL-4 ↑	[35]
Human PBMC *	Cocoa procyanidins (monomers through decamers; 25 μg/mL)	TNF-α secretion ↑	[37]
Human PBMC *	Cocoa procyanidins (monomers through decamers; 25 μg/mL)	IL-5 secretion ↑ (monomers-trimers) IL-5 secretion ↓ (hexamers-decamers)	[38]
Human PBMC°	Short- (monomers-pentamers) & long-chain (hexamers-decamers) flavanol fractions (20 μg/mL)	Inflammatory mediators (IL-1β, IL-6, IL-10, TNF-α) ↑	[53]
Whole blood	Purified trimeric & pentameric cocoa procyanidins (3, 10 μmol/L)	PAC-1 binding & P-selectin expression ↑ in unstimulated platelets Epinephrine-induced platelet activation ↓	[54]
Isolated rabbit aortic rings	Procyanidin-rich cocoa extracts	Endothelium-dependent relaxation ↑ NOS activity ↑	[45]
ACE from rabbit lung	Epicatechin, dimeric & hexameric procyanidins (0–500 μmol/L)	ACE activity ↓ molecular weight-dependent	[47]
Murine EL4BOU6 lymphocytes	Cocoa extract (5–80 μg/mL total polyphenols) vs. epicatechin (60–120 μg/mL total polyphenols)	IL-2 secretion ↓ IL-4 secretion↑ T lymphocyte activation ↓	[41]
Murine RAW264.7 macrophages # Rat NR8383 macrophages #	Cocoa extract (5–100 μg/mL total polyphenols) vs. epicatechin (60–120 μg/mL total polyphenols) Cocoa extract (10–50 μg/mL total polyphenols) vs. epicatechin (30–60 μg/mL total polyphenols)	inducible NO ↓ Proinflammatory mediators (TNF-α, MCP-1, IL-1α, IL-6) ↓	[42]

Jurkat T cells	Catechin, epicatechin & B-type oligomers (1.7–17.2 μmol/L)	PMA-induced NF-κB activation ↓ IL-2 expression & secretion ↓	[55]
VSMC	Cocoa procyanidins (0–100 μg/mL) & procyanidin B2 (0–100 μmol/L)	MMP-2 expression & activation ↓ VSMC invasion & migration ↓ MT1-MMP & MEk1 activities ↓	[46]
HUVEC	Epicatechin & flavanol metabolites mixture vs. control	Arginase-2 mRNA expression ↓ Arginase activity ↓ dose-dependent	[44]

GAE gallic acid equivalents,

° PBMC, peripheral blood mononuclear cells;

*, phytohemagglutinin (PHA)-stimulated;

#, lipopolysaccharide (LPS)-stimulated; MCP-1, monocyte chemoattractant protein-1;

†, 12-O-tetradecaoylphorbol-13-acetate (TPA)-stimulated; LOX, lipoxygenase; COX-2, cyclooxygenase-2; VSMC, vascular smooth muscle cells; MMP, matrix metalloproteinase; MT1, membrane type-1; MEK, mitogen-activated protein kinase kinase; HUVEC, human umbilical endothelial cells.

**Table 3.** Potential mechanisms by which polyphenols from cocoa may affect vascular health.

- Inhibition of LDL oxidation - Inhibition of lipoxygenase activity - Inhibition of inflammatory gene expression (e.g., IL1β, IL2, IL4, IL6, TNFα, iNOS) due to inhibition of NFκB and AP1 transcription factor activity - Inhibition of the expression of genes encoding for cell adhesion proteins, chemotactic factors and metalloproteinases - Increase in endothelial nitric oxide synthase activity - Inhibition of arginase activity - Inhibition of platelet aggregation - Increase in HDL and decrease in LDL and triglyceride levels

**Table 4.** *In vivo* studies with cocoa polyphenols in laboratory animals.

**Species**	**No. animals**	**Experimental duration (wk)**	**Treatment**	**Outcomes**	**Reference**
Rats	5	–	Oral administration of 1 g cocoa powder/kg bw (7.8 mg epicatechin)	Lipid peroxides in plasma ↓ Oxidant-induced α-tocopherol consumption ↓	[4]
Rats	5–6	–	Gastric intubation with 100 mg cocoa extract	Plasma antioxidant capacity ↑ Erythrocyte hemolysis ↓	[56]
Rats	48	2	Diet containing by weight 0.5–2.0% flavanol- and procyanidin-rich cocoa (12.2 mg/g epicatechin, 2.8 mg/g catechin, 53.3 mg/g procyanidins)	Oxidative DNA damage in testes ↓ No effect on plasma F_2_-isoprostane and TBARS	[52]
Hypercholesterolemic rabbits	12	24	Diet containing 10% cocoa powder (0.78 g total polyphenols)	TBARS in plasma↓ No effects on plasma cholesterol, TG & phospholipids LDL oxidation lag time ↑ Area of atherosclerotic lesions in aorta ↓	[48]
Hamsters	27	10	Brownie (10 g vs. 1 g cocoa powder)	LDL & TG levels ↓ HDL ↑ dose-dependent LDL-oxidizability ↓	[49]
Obese-diabetic rats	40	4	Cocoa extract (600 mg/kg bw/d)	Blood glucose levels ↓ Plasma free fatty acids ↓ 8-isoprostane levels ↓ Superoxide dismutase activity ↑ No change in catalase activity	[51]
Rats	10	4	40 g cocoa powder per kg diet (11.0 mg/g epicatechin, 2.8 mg/g catechin, 43.0 mg/g procyanidins), vs. none	Renal arginase activity ↓	[44]

TG, triglycerides, TBARS: Thiobarbituric acid reactive substances.

**Table 5.** Human intervention trials with cocoa and chocolate.

**No. Subjects**	**Age range (mean)**	**BMI (kg/m^2^)**	**Intervention**	**Polyphenol content**	**Outcomes**	**Reference**
12	39 ± 4.0	–	Cocoa	–	LDL oxidation ↓	[57]
15	(32.5 ± 6.4)	21.7 ± 2.1	12 g cocoa powder x3/d for 2 weeks, vs. sugar	2610 mg total polyphenols/d (244 mg epicatechin)	LDL oxidation ↓ No change in plasma lipids or antioxidants Urinary excretion of epicatechin/metabolites ↑	[58]
23	21–62 (36)	–	22 g cocoa powder + 16 g dark chocolate/d for 4 weeks, vs. average American diet	466 mg procyanidins/d (111 mg monomers)	LDL oxidation ↓ Serum antioxidant capacity ↑ HDL cholesterol ↑	[19]
25	20–6 (32.4 ± 7.4)	24.4 ± 3.4	37 g dark chocolate & 31 g cocoa powder in a drink/d for 6 weeks, vs. none	651 mg total procyanidins/d (chocolate = 168 mg/d, cocoa = 483 mg/d)	LDL oxidizability ↓ No effect on inflammation markers, or plasma antioxidant capacity	[81]
45	19–49 (26)	21.5 ± 2.9 /24.1 ± 3.5	75 g dark chocolate or high-phenolic dark chocolate for 3 weeks, vs. 75 g white chocolate	Total polyphenols (epicatechin): Dark = 274 (114) mg/d High = 418 (170) mg/d	HDL cholesterol ↑ Lipid peroxidation ↓ No change in plasma antioxidant capacity	[60]
25	(38 ± 1)	22.1 ± 0.4	26 g/d cocoa powder for 12 weeks	*Per 100 g:* 377 mg epicatechin, 135 mg catechin, 158 mg procyanidin B2, 96.1 mg procyanidin C1	LDL oxidation ↓ HDL-cholesterol ↑	[59]
20	20–56	23.8 ± 0.79	Semi-sweet chocolate baking bits (27, 53, 80 g), vs. none	Total procyanidins (epicatechin): 186 (46) mg 365 (90) mg 551 (136) mg	Plasma epicatechin ↑ dose-dependent Antioxidant capacity ↑ TBARS ↓	[17]
13	26–49	23.2 ± 1.2	105 g (of which 80 g chocolate) semi-sweet baking bits, vs. vanilla milk chips	557 mg total procyanidins (137 mg epicatechin)	Plasma epicatechin ↑ Total antioxidant capacity ↑ TBARS ↓	[10]
20	20–40	–	100 ml high- vs. low-flavanol cocoa drink	187 mg vs. 14 mg total monomers & oligomeric procyanidins	Plasma F2-isoprostanes ↓	[62]
12	25–35 (32.2 ± 1.0)	21.9 ± 0.4	100 g dark chocolate (with & without 200 mL milk), vs. 200 g milk chocolate	Not stated but FRAP values (147.4 μmol FE/100 g)	Plasma antioxidant capacity & epicatechin ↑, in absence of milk	[9]
30	24–49	–	Cocoa beverage (300 ml, 18.75 g procyanidin-rich cocoa powder), caffeinated beverage, or water	897 mg total epicatechin & oligomeric procyanidins	Platelet activation & function ↓	[61]
18	–	–	25 g semi-sweet chocolate chips, vs. none	220 mg flavanols & procyanidins	Plasma epicatechin ↑ Platelet function ↓	[24]
32	40 ± 9	26 ± 4	Cocoa flavanol/procyanidin tablets for 28 d, vs. placebo	234 mg flavanols & procyanidins/d	Platelet aggregation ↓ Plasma ascorbic acid ↑ No change in oxidation status markers Plasma epicatechin & catechin ↑	[25]
30	20–58 (30.6)	–	Dark (75% cocoa), vs. milk (20% cocoa) or white (no flavonoids) chocolate High polyphenol vs. low	–	Collagen–induced platelet aggregation ↓	[82]
27	18–72 (44 ± 3.4)	26.9 ± 0.9	flavanol cocoa drink (4x 230 mL/d for 4 d)	821 mg/d total flavanols (epicatechin, catechin & related oligomers)	Improved peripheral vasodilation	[63]
20	41 ± 14	25 ± 4	100 mL high polyphenol vs. low flavanol cocoa drink	176 mg total flavanols (70 mg monomers, 106 mg procyanidins)	NO bioactivity ↑ Arterial FMD ↑	[64]
10	–	–	200 mL high- vs. low-flavanol cocoa beverage	985 vs. 80.4 mg total flavanols	Erythrocyte arginase activity ↓	[44]
13	55–64	21.9–26.2	100 g dark chocolate/d for 14 d, vs. 90 g white chocolate in hypertensive subjects	500 mg/d total polyphenols	Systolic & diastolic blood pressure ↓	[72]
15	(33.9 ± 7.6)	22.6 ± 3.0	100 g dark chocolate, vs. 90 g white chocolate for 15 d	500 mg total polyphenols	Insulin sensitivity ↑ Insulin resistance ↓ Systolic blood pressure ↓	[70]
28	–	–	105 g/d milk chocolate for 14 d, vs. cocoa butter chocolate; hypertensive subjects	168 mg/d flavanols (39 mg monomers, 126 mg polymers) 2.62 g procyanidins	Diastolic & mean blood pressure ↓ LDL cholesterol ↓ Oxidative stress markers ↓	[69]
17	24–32 (28.9)	<27.0	100 g dark chocolate, vs. none	(0.54 g monomers & dimers, 0.76 g trimer-heptamers)	Improved endothelial function Vasodilation of brachial artery No change in blood pressure	[67]
20	(43.65 ± 7.8)	25.4 ± 1.7	100 g/d dark chocolate for 15 d, vs. 90 g white chocolate in hypertensive subjects	88 mg/d flavanols (22 mg catechin, 66 mg epicatechin)	Improved insulin sensitivity Systolic & diastolic blood pressure ↓ LDL cholesterol ↓ Improved FMD	[71]
11	(31 ± 1)	21.8 ± 0.8	100 mL high- vs. low-polyphenol cocoa drink	176–185 mg flavanols (70–74 mg monomers, 20–22 mg epicatechin, 106–111 mg procyanidins)	Circulating NO & FMD ↑	[65]
16	25–32	19–23	300 mL high- vs. low-flavanol cocoa drink	917 mg flavanols (19% epicatechin)	Circulating NO species ↑ FMD response of conduit arteries ↑ Microcirculation ↑	[66]
20	–	–	40 g dark chocolate, vs. white chocolate	Same brand as used for Vlachopoulos *et al*. (2005)	Improved FMD Platelet function ↓ Plasma total antioxidant status ↑	[83]
34	18–74 (47.9 ± 3.0)	28.0 ± 1.9/28.4 ± 1.3	High polyphenol cocoa drink 4x 230 mL/d for 4–6 d, vs. none	*Per 100 mL*: 9.2 mg epicatechin, 10.7 mg catechin, 69.3 mg flavanol oligomers (821 mg/d)	NO synthesis ↓ FMD ↑ Pulse wave amplitude ↑	[84]
40	61 ± 9	27.1 ± 3.9	48 g flavanol-rich chocolate bar + 18 g cocoa beverage/d, vs. placebo for 6 weeks in subjects with coronary artery disease	444 vs. 19.6 mg/d total flavanols (107 vs. 4.7 mg epicatechin)	No acute or chronic changes in FMD, systemic arterial compliance, forearm blood flow, soluble cellular adhesion molecules	[75]
32	57.7 ± 2.2/55.4 ± 1.7	24.9 ± 1.0/25.3 ± 0.8	High- vs. low-flavanol cocoa beverage for 6 weeks in hypercholesterolemic subjects	446 vs. 43 mg total flavanols	FMD ↑ Brachial artery hyperaemic blood flow ↑ VCAM-1 ↓	[85]
11	22–32 (27 ± 1)	22 ± 1	100 mL high-flavanol vs. low-phenolic cocoa drink x3/d for 1 week	*Per 100 ml*: 59 mg epicatechin, 15 mg catechin, 232 mg flavanol oligomers (918 mg/d procyanidins)	FMD ↑ No change in biomarkers of oxidative stress	[86]
45	30–75 (52.8 11.0) ±	30.1 ± 3.3	74 g solid dark chocolate (22 g cocoa powder); 240 ml liquid cocoa (sugar-free vs. sugared)	821 mg total flavanols; 805.2 & 8.5 mg total flavanols	Improved FMD Systolic & diastolic blood pressure ↓	[68]

FE: Ferric equivalents; FMD: Flow-mediated dilation; FRAP: Ferric-reducing ability of plasma;

TBARS: Thiobarbituric acid reactive substances.

All data refer to healthy subjects unless otherwise stated.

## References

[1] Visioli F Bernaert H Corti R Ferri C Heptinstall S Molinari E Poli A Serafini M Smit HJ Vinson JA Violi F Paoletti R Chocolate, lifestyle, and health Crit. Rev. Food Sci. Nutr 2009 49 299 312 19234942 10.1080/10408390802066805

[2] Galleano M Oteiza PI Fraga CG Cocoa, chocolate and cardiovascular disease J Cardiovasc Pharmacol 2009DOI: 10.1097/FJC.0b013e3181b76787. 10.1097/FJC.0b013e3181b76787 PMC2797556 19701098

[3] Corti R Flammer AJ Hollenberg NK Luscher TF Cocoa and cardiovascular health Circulation 2009 119 1433 1441 19289648 10.1161/CIRCULATIONAHA.108.827022

[4] Baba S Osakabe N Natsume M Yasuda A Takizawa T Nakamura T Terao J Cocoa powder enhances the level of antioxidative activity in rat plasma Br. J. Nutr 2000 84 673 680 11177180

[5] Andres-Lacueva C Monagas M Khan N Izquierdo-Pulido M Urpi-Sarda M Permanyer J Lamuela-Raventos RM Flavanol and flavonol contents of cocoa powder products: Influence of the manufacturing process J. Agric. Food Chem 2008 56 3111 3117 18412367 10.1021/jf0728754

[6] Grassi D Desideri G Necozione S Lippi C Casale R Properzi G Blumberg JB Ferri C Blood pressure is reduced and insulin sensitivity increased in glucose-intolerant, hypertensive subjects after 15 days of consuming high-polyphenol dark chocolate J. Nutr 2008 138 1671 1676 18716168 10.1093/jn/138.9.1671

[7] Hammerstone JF Lazarus SA Mitchell AE Rucker R Schmitz HH Identification of procyanidins in cocoa (Theobroma cacao) and chocolate using high-performance liquid chromatography/mass spectrometry J. Agric. Food Chem 1999 47 490 496 10563922 10.1021/jf980760h

[8] Ramiro-Puig E Castell M Cocoa: Antioxidant and immunomodulator Br. J. Nutr 2009 101 931 940 19126261 10.1017/S0007114508169896

[9] Serafini M Bugianesi R Maiani G Valtuena S de Santis S Crozier A Plasma antioxidants from chocolate Nature 2003 424 1013 12944955 10.1038/4241013a

[10] Rein D Lotito S Holt RR Keen CL Schmitz HH Fraga CG Epicatechin in human plasma: *In vivo* determination and effect of chocolate consumption on plasma oxidation status J. Nutr 2000 130 2109S 2114S 10917931 10.1093/jn/130.8.2109S

[11] Donovan JL Crespy V Oliveira M Cooper KA Gibson BB Williamson G (+)-Catechin is more bioavailable than (−)-catechin: Relevance to the bioavailability of catechin from cocoa Free Radic. Res 2006 40 1029 1034 17015247 10.1080/10715760600868545

[12] Roura E Andres-Lacueva C Estruch R Mata-Bilbao ML Izquierdo-Pulido M Waterhouse AL Lamuela-Raventos RM Milk does not affect the bioavailability of cocoa powder flavonoid in healthy human Ann. Nutr. Metab 2007 51 493 498 18032884 10.1159/000111473

[13] Schramm DD Karim M Schrader HR Holt RR Kirkpatrick NJ Polagruto JA Ensunsa JL Schmitz HH Keen CL Food effects on the absorption and pharmacokinetics of cocoa flavanols Life Sci 2003 73 857 869 12798412 10.1016/s0024-3205(03)00373-4

[14] Crozier A Jaganath IB Clifford MN Dietary phenolics: Chemistry, bioavailability and effects on health Nat. Prod. Rep 2009 26 1001 1043 19636448 10.1039/b802662a

[15] Steinberg FM Bearden MM Keen CL Cocoa and chocolate flavonoids: Implications for cardiovascular health J. Am. Diet. Assoc 2003 103 215 223 12589329 10.1053/jada.2003.50028

[16] Richelle M Tavazzi I Enslen M Offord EA Plasma kinetics in man of epicatechin from black chocolate Eur. J. Clin. Nutr 1999 53 22 26 10048796 10.1038/sj.ejcn.1600673

[17] Wang JF Schramm DD Holt RR Ensunsa JL Fraga CG Schmitz HH Keen CL A dose-response effect from chocolate consumption on plasma epicatechin and oxidative damage J. Nutr 2000 130 2115S 2119S 10917932 10.1093/jn/130.8.2115S

[18] Baba S Osakabe N Yasuda A Natsume M Takizawa T Nakamura T Terao J Bioavailability of (−)-epicatechin upon intake of chocolate and cocoa in human volunteers Free Radic. Res 2000 33 635 641 11200094 10.1080/10715760000301151

[19] Wan Y Vinson JA Etherton TD Proch J Lazarus SA Kris-Etherton PM Effects of cocoa powder and dark chocolate on LDL oxidative susceptibility and prostaglandin concentrations in humans Am. J. Clin. Nutr 2001 74 596 602 11684527 10.1093/ajcn/74.5.596

[20] Schramm DD Wang JF Holt RR Ensunsa JL Gonsalves JL Lazarus SA Schmitz HH German JB Keen CL Chocolate procyanidins decrease the leukotriene-prostacyclin ratio in humans and human aortic endothelial cells Am. J. Clin. Nutr 2001 73 36 40 11124747 10.1093/ajcn/73.1.36

[21] Pearson DA Paglieroni TG Rein D Wun T Schramm DD Wang JF Holt RR Gosselin R Schmitz HH Keen CL The effects of flavanol-rich cocoa and aspirin on ex vivo platelet function Thromb. Res 2002 106 191 197 12297125 10.1016/s0049-3848(02)00128-7

[22] Holt RR Lazarus SA Sullards MC Zhu QY Schramm DD Hammerstone JF Fraga CG Schmitz HH Keen CL Procyanidin dimer B2 [epicatechin-(4beta-8)-epicatechin] in human plasma after the consumption of a flavanol-rich cocoa Am. J. Clin. Nutr 2002 76 798 804 12324293 10.1093/ajcn/76.4.798

[23] Steinberg FM Holt RR Schmitz HH Keen CL Cocoa procyanidin chain length does not determine ability to protect LDL from oxidation when monomer units are controlled J. Nutr. Biochem 2002 13 645 652 12550061 10.1016/s0955-2863(02)00215-2

[24] Holt RR Schramm DD Keen CL Lazarus SA Schmitz HH Chocolate consumption and platelet function JAMA 2002 287 2212 2213 11980520 10.1001/jama.287.17.2212

[25] Murphy KJ Chronopoulos AK Singh I Francis MA Moriarty H Pike MJ Turner AH Mann NJ Sinclair AJ Dietary flavanols and procyanidin oligomers from cocoa (Theobroma cacao) inhibit platelet function Am. J. Clin. Nutr 2003 77 1466 1473 12791625 10.1093/ajcn/77.6.1466

[26] Waterhouse AL Shirley JR Donovan JL Antioxidants in chocolate Lancet 1996 348 834 8814019 10.1016/S0140-6736(05)65262-2

[27] Lotito SB Actis-Goretta L Renart ML Caligiuri M Rein D Schmitz HH Steinberg FM Keen CL Fraga CG Influence of oligomer chain length on the antioxidant activity of procyanidins Biochem. Biophys. Res. Commun 2000 276 945 951 11027573 10.1006/bbrc.2000.3571

[28] Richelle M Tavazzi I Offord E Comparison of the antioxidant activity of commonly consumed polyphenolic beverages (coffee, cocoa, and tea) prepared per cup serving J. Agric. Food Chem 2001 49 3438 3442 11453788 10.1021/jf0101410

[29] Osakabe N Yasuda A Natsume M Takizawa T Terao J Kondo K Catechins and their oligomers linked by C4 → C8 bonds are major cacao polyphenols and protect low-density lipoprotein from oxidation *in vitro* Exp. Biol. Med. (Maywood) 2002 227 51 56 11788784 10.1177/153537020222700109

[30] Vinson JA Proch J Zubik L Phenol antioxidant quantity and quality in foods: Cocoa, dark chocolate, and milk chocolate J. Agric. Food Chem 1999 47 4821 4824 10606537 10.1021/jf990312p

[31] Schewe T Sadik C Klotz LO Yoshimoto T Kuhn H Sies H Polyphenols of cocoa: Inhibition of mammalian 15-lipoxygenase Biol. Chem 2001 382 1687 1696 11843182 10.1515/BC.2001.204

[32] Schewe T Kuhn H Sies H Flavonoids of cocoa inhibit recombinant human 5-lipoxygenase J. Nutr 2002 132 1825 1829 12097654 10.1093/jn/132.7.1825

[33] Hatano T Miyatake H Natsume M Osakabe N Takizawa T Ito H Yoshida T Proanthocyanidin glycosides and related polyphenols from cacao liquor and their antioxidant effects Phytochemistry 2002 59 749 758 11909632 10.1016/s0031-9422(02)00051-1

[34] Mao T van de Water J Keen CL Schmitz HH Gershwin ME Cocoa procyanidins and human cytokine transcription and secretion J. Nutr 2000 130 2093S 2099S 10917928 10.1093/jn/130.8.2093S

[35] Mao TK Powell J van de Water J Keen CL Schmitz HH Gershwin ME Effect of cocoa procyanidins on the secretion of interleukin-4 in peripheral blood mononuclear cells J. Med. Food 2000 3 107 114 10.1016/s0024-3205(00)00449-5 11210713

[36] Mao TK Powell J van de Water J Keen CL Schmitz HH Hammerstone JF Gershwin ME The effect of cocoa procyanidins on the transcription and secretion of interleukin 1 beta in peripheral blood mononuclear cells Life Sci 2000 66 1377 1386 11210713 10.1016/s0024-3205(00)00449-5

[37] Mao TK van de Water J Keen CL Schmitz HH Gershwin ME Modulation of TNF-alpha secretion in peripheral blood mononuclear cells by cocoa flavanols and procyanidins Dev. Immunol 2002 9 135 141 12885154 10.1080/1044667031000137601 PMC2276101

[38] Mao TK van de Water J Keen CL Schmitz HH Gershwin ME Effect of cocoa flavanols and their related oligomers on the secretion of interleukin-5 in peripheral blood mononuclear cells J. Med. Food 2002 5 17 22 12511109 10.1089/109662002753723188

[39] Guo Q Rimbach G Packer L Nitric oxide formation in macrophages detected by spin trapping with iron-dithiocarbamate complex: Effect of purified flavonoids and plant extracts Methods Enzymol 2001 335 273 282 11400376 10.1016/s0076-6879(01)35250-3

[40] Saliou C Valacchi G Rimbach G Assessing bioflavonoids as regulators of NF-kappa B activity and inflammatory gene expression in mammalian cells Methods Enzymol 2001 335 380 387 11400387 10.1016/s0076-6879(01)35260-6

[41] Ramiro E Franch A Castellote C Andres-Lacueva C Izquierdo-Pulido M Castell M Effect of Theobroma cacao flavonoids on immune activation of a lymphoid cell line Br. J. Nutr 2005 93 859 866 16022755 10.1079/bjn20051443

[42] Ramiro E Franch A Castellote C Perez-Cano F Permanyer J Izquierdo-Pulido M Castell M Flavonoids from Theobroma cacao down-regulate inflammatory mediators J. Agric. Food Chem 2005 53 8506 8511 16248545 10.1021/jf0511042

[43] Park YC Rimbach G Saliou C Valacchi G Packer L Activity of monomeric, dimeric, and trimeric flavonoids on NO production, TNF-alpha secretion, and NF-kappaB-dependent gene expression in RAW 264.7 macrophages FEBS Lett 2000 465 93 97 10631311 10.1016/s0014-5793(99)01735-4

[44] Schnorr O Brossette T Momma TY Kleinbongard P Keen CL Schroeter H Sies H Cocoa flavanols lower vascular arginase activity in human endothelial cells *in vitro* and in erythrocytes *in vivo* Arch. Biochem. Biophys 2008 476 211 215 18348861 10.1016/j.abb.2008.02.040

[45] Karim M McCormick K Kappagoda CT Effects of cocoa extracts on endothelium-dependent relaxation J. Nutr 2000 130 2105S 2108S 10917930 10.1093/jn/130.8.2105S

[46] Lee KW Kang NJ Oak MH Hwang MK Kim JH Schini-Kerth VB Lee HJ Cocoa procyanidins inhibit expression and activation of MMP-2 in vascular smooth muscle cells by direct inhibition of MEK and MT1-MMP activities Cardiovasc. Res 2008 79 34 41 18310679 10.1093/cvr/cvn056

[47] Actis-Goretta L Ottaviani JI Keen CL Fraga CG Inhibition of angiotensin converting enzyme (ACE) activity by flavan-3-ols and procyanidins FEBS Lett 2003 555 597 600 14675780 10.1016/s0014-5793(03)01355-3

[48] Kurosawa T Itoh F Nozaki A Nakano Y Katsuda S Osakabe N Tsubone H Kondo K Itakura H Suppressive effect of cocoa powder on atherosclerosis in Kurosawa and Kusanagi-hypercholesterolemic rabbits J. Atheroscler. Thromb 2005 12 20 28 15725692 10.5551/jat.12.20

[49] Vinson JA Proch J Bose P Muchler S Taffera P Shuta D Samman N Agbor GA Chocolate is a powerful *ex vivo* and *in vivo* antioxidant, an antiatherosclerotic agent in an animal model, and a significant contributor to antioxidants in the European and American Diets J. Agric. Food Chem 2006 54 8071 8076 17032011 10.1021/jf062175j

[50] Yasuda A Natsume M Sasaki K Baba S Nakamura Y Kanegae M Nagaoka S Cacao procyanidins reduce plasma cholesterol and increase fecal steroid excretion in rats fed a high-cholesterol diet Biofactors 2008 33 211 223 19478425 10.1002/biof.5520330307

[51] Jalil AM Ismail A Pei CP Hamid M Kamaruddin SH Effects of cocoa extract on glucometabolism, oxidative stress, and antioxidant enzymes in obese-diabetic (ob-db) rats J. Agric. Food Chem 2008 56 7877 7884 18702467 10.1021/jf8015915

[52] Orozco TJ Wang JF Keen CL Chronic consumption of a flavanol- and procyanindin-rich diet is associated with reduced levels of 8-hydroxy-2′-deoxyguanosine in rat testes J. Nutr. Biochem 2003 14 104 110 12667602 10.1016/s0955-2863(02)00273-5

[53] Kenny TP Keen CL Schmitz HH Gershwin ME Immune effects of cocoa procyanidin oligomers on peripheral blood mononuclear cells Exp. Biol. Med. (Maywood) 2007 232 293 300 17259337

[54] Rein D Paglieroni TG Pearson DA Wun T Schmitz HH Gosselin R Keen CL Cocoa and wine polyphenols modulate platelet activation and function J. Nutr 2000 130 2120S 2126S 10917933 10.1093/jn/130.8.2120S

[55] Mackenzie GG Carrasquedo F Delfino JM Keen CL Fraga CG Oteiza PI Epicatechin, catechin, and dimeric procyanidins inhibit PMA-induced NF-kappaB activation at multiple steps in Jurkat T cells FASEB J 2004 18 167 169 14630700 10.1096/fj.03-0402fje

[56] Zhu QY Holt RR Lazarus SA Orozco TJ Keen CL Inhibitory effects of cocoa flavanols and procyanidin oligomers on free radical-induced erythrocyte hemolysis Exp. Biol. Med. (Maywood) 2002 227 321 329 11976402 10.1177/153537020222700504

[57] Kondo K Hirano R Matsumoto A Igarashi O Itakura H Inhibition of LDL oxidation by cocoa Lancet 1996 348 1514 8942794 10.1016/s0140-6736(05)65927-2

[58] Osakabe N Baba S Yasuda A Iwamoto T Kamiyama M Takizawa T Itakura H Kondo K Daily cocoa intake reduces the susceptibility of low-density lipoprotein to oxidation as demonstrated in healthy human volunteers Free Radic. Res 2001 34 93 99 11235000 10.1080/10715760100300091

[59] Baba S Osakabe N Kato Y Natsume M Yasuda A Kido T Fukuda K Muto Y Kondo K Continuous intake of polyphenolic compounds containing cocoa powder reduces LDL oxidative susceptibility and has beneficial effects on plasma HDL-cholesterol concentrations in humans Am. J. Clin. Nutr 2007 85 709 717 17344491 10.1093/ajcn/85.3.709

[60] Mursu J Voutilainen S Nurmi T Rissanen TH Virtanen JK Kaikkonen J Nyyssonen K Salonen JT Dark chocolate consumption increases HDL cholesterol concentration and chocolate fatty acids may inhibit lipid peroxidation in healthy humans Free Radic. Biol. Med 2004 37 1351 1359 15454274 10.1016/j.freeradbiomed.2004.06.002

[61] Rein D Paglieroni TG Wun T Pearson DA Schmitz HH Gosselin R Keen CL Cocoa inhibits platelet activation and function Am. J. Clin. Nutr 2000 72 30 35 10871557 10.1093/ajcn/72.1.30

[62] Wiswedel I Hirsch D Kropf S Gruening M Pfister E Schewe T Sies H Flavanol-rich cocoa drink lowers plasma F(2)-isoprostane concentrations in humans Free Radic. Biol. Med 2004 37 411 421 15223075 10.1016/j.freeradbiomed.2004.05.013

[63] Fisher ND Hughes M Gerhard-Herman M Hollenberg NK Flavanol-rich cocoa induces nitric-oxide-dependent vasodilation in healthy humans J. Hypertens 2003 21 2281 2286 14654748 10.1097/00004872-200312000-00016

[64] Heiss C Dejam A Kleinbongard P Schewe T Sies H Kelm M Vascular effects of cocoa rich in flavan-3-ols JAMA 2003 290 1030 1031 12941674 10.1001/jama.290.8.1030

[65] Heiss C Kleinbongard P Dejam A Perre S Schroeter H Sies H Kelm M Acute consumption of flavanol-rich cocoa and the reversal of endothelial dysfunction in smokers J. Am. Coll. Cardiol 2005 46 1276 1283 16198843 10.1016/j.jacc.2005.06.055

[66] Schroeter H Heiss C Balzer J Kleinbongard P Keen CL Hollenberg NK Sies H Kwik-Uribe C Schmitz HH Kelm M (−)-Epicatechin mediates beneficial effects of flavanol-rich cocoa on vascular function in humans Proc. Natl. Acad. Sci. USA 2006 103 1024 1029 16418281 10.1073/pnas.0510168103 PMC1327732

[67] Vlachopoulos C Aznaouridis K Alexopoulos N Economou E Andreadou I Stefanadis C Effect of dark chocolate on arterial function in healthy individuals Am J Hypertens 2005 18 785 791 15925737 10.1016/j.amjhyper.2004.12.008

[68] Faridi Z Njike VY Dutta S Ali A Katz DL Acute dark chocolate and cocoa ingestion and endothelial function: A randomized controlled crossover trial Am. J. Clin. Nutr 2008 88 58 63 18614724 10.1093/ajcn/88.1.58

[69] Fraga CG Cocoa, diabetes, and hypertension: Should we eat more chocolate? Am. J. Clin. Nutr 2005 81 541 542 15755820 10.1093/ajcn/81.3.541

[70] Grassi D Lippi C Necozione S Desideri G Ferri C Short-term administration of dark chocolate is followed by a significant increase in insulin sensitivity and a decrease in blood pressure in healthy persons Am. J. Clin. Nutr 2005 81 611 614 15755830 10.1093/ajcn/81.3.611

[71] Grassi D Necozione S Lippi C Croce G Valeri L Pasqualetti P Desideri G Blumberg JB Ferri C Cocoa reduces blood pressure and insulin resistance and improves endothelium-dependent vasodilation in hypertensives Hypertension 2005 46 398 405 16027246 10.1161/01.HYP.0000174990.46027.70

[72] Taubert D Berkels R Roesen R Klaus W Chocolate and blood pressure in elderly individuals with isolated systolic hypertension JAMA 2003 290 1029 1030 12941673 10.1001/jama.290.8.1029

[73] Taubert D Roesen R Lehmann C Jung N Schomig E Effects of low habitual cocoa intake on blood pressure and bioactive nitric oxide: A randomized controlled trial JAMA 2007 298 49 60 17609490 10.1001/jama.298.1.49

[74] Balzer J Rassaf T Heiss C Kleinbongard P Lauer T Merx M Heussen N Gross HB Keen CL Schroeter H Kelm M Sustained benefits in vascular function through flavanol-containing cocoa in medicated diabetic patients a double-masked, randomized, controlled trial J. Am. Coll. Cardiol 2008 51 2141 2149 18510961 10.1016/j.jacc.2008.01.059

[75] Farouque HM Leung M Hope SA Baldi M Schechter C Cameron JD Meredith IT Acute and chronic effects of flavanol-rich cocoa on vascular function in subjects with coronary artery disease: A randomized double-blind placebo-controlled study Clin. Sci. (Lond) 2006 111 71 80 16551272 10.1042/CS20060048

[76] Mehrinfar R Frishman WH Flavanol-rich cocoa: A cardioprotective nutraceutical Cardiol. Rev 2008 16 109 115 18414181 10.1097/CRD.0b013e31815d95e2

[77] Flammer AJ Hermann F Sudano I Spieker L Hermann M Cooper KA Serafini M Luscher TF Ruschitzka F Noll G Corti R Dark chocolate improves coronary vasomotion and reduces platelet reactivity Circulation 2007 116 2376 2382 17984375 10.1161/CIRCULATIONAHA.107.713867

[78] Turner R Baron T Wolffram S Minihane AM Cassidy A Rimbach G Weinberg PD Effect of circulating forms of soy isoflavones on the oxidation of low density lipoprotein Free Radic. Res 2004 38 209 216 15104215 10.1080/10715760310001641854

[79] Rimbach G Weinberg PD de Pascual-Teresa S Alonso MG Ewins BA Turner R Minihane AM Botting N Fairley B Matsugo S Uchida Y Cassidy A Sulfation of genistein alters its antioxidant properties and its effect on platelet aggregation and monocyte and endothelial function Biochim. Biophys. Acta 2004 1670 229 237 14980449 10.1016/j.bbagen.2003.12.008

[80] Halliwell B Plasma antioxidants: Health benefits of eating chocolate Nature 2003 426 787discussion 788. 14685224 10.1038/426787a

[81] Mathur S Devaraj S Grundy SM Jialal I Cocoa products decrease low density lipoprotein oxidative susceptibility but do not affect biomarkers of inflammation in humans J. Nutr 2002 132 3663 3667 12468604 10.1093/jn/132.12.3663

[82] Innes AJ Kennedy G McLaren M Bancroft AJ Belch JJ Dark chocolate inhibits platelet aggregation in healthy volunteers Platelets 2003 14 325 327 12944249 10.1080/0953710031000123681

[83] Hermann F Spieker LE Ruschitzka F Sudano I Hermann M Binggeli C Luscher TF Riesen W Noll G Corti R Dark chocolate improves endothelial and platelet function Heart 2006 92 119 120 16365364 10.1136/hrt.2005.063362 PMC1860996

[84] Fisher ND Hollenberg NK Aging and vascular responses to flavanol-rich cocoa J. Hypertens 2006 24 1575 1580 16877960 10.1097/01.hjh.0000239293.40507.2a

[85] Wang-Polagruto JF Villablanca AC Polagruto JA Lee L Holt RR Schrader HR Ensunsa JL Steinberg FM Schmitz HH Keen CL Chronic consumption of flavanol-rich cocoa improves endothelial function and decreases vascular cell adhesion molecule in hypercholesterolemic postmenopausal women J Cardiovasc Pharmacol 2006 47 Suppl. 2 S177 S186discussion S206–S209. 16794456 10.1097/00005344-200606001-00013

[86] Heiss C Finis D Kleinbongard P Hoffmann A Rassaf T Kelm M Sies H Sustained increase in flow-mediated dilation after daily intake of high-flavanol cocoa drink over 1 week J. Cardiovasc. Pharmacol 2007 49 74 80 17312446 10.1097/FJC.0b013e31802d0001

[87] Mohan S Campbell NR Salt and high blood pressure Clin. Sci. (Lond) 2009 117 1 11 19476440 10.1042/CS20080207

[88] Davison K Coates AM Buckley JD Howe PR Effect of cocoa flavanols and exercise on cardiometabolic risk factors in overweight and obese subjects Int. J. Obes. (Lond) 2008 32 1289 1296 18504447 10.1038/ijo.2008.66

[89] Ferri C Grassi G Mediterranean diet, cocoa and cardiovascular disease: A sweeter life, a longer life, or both? J. Hypertens 2003 21 2231 2234 14654738 10.1097/00004872-200312000-00006

[90] Grassi D Desideri G Croce G Tiberti S Aggio A Ferri C Flavonoids, vascular function and cardiovascular protection Curr. Pharm. Des 2009 15 1072 1084 19355949 10.2174/138161209787846982

[91] Joshipura KJ Hu FB Manson JE Stampfer MJ Rimm EB Speizer FE Colditz G Ascherio A Rosner B Spiegelman D Willett WC The effect of fruit and vegetable intake on risk for coronary heart disease Ann. Intern. Med 2001 134 1106 1114 11412050 10.7326/0003-4819-134-12-200106190-00010

[92] Hertog MG Feskens EJ Hollman PC Katan MB Kromhout D Dietary antioxidant flavonoids and risk of coronary heart disease: The Zutphen elderly study Lancet 1993 342 1007 1011 8105262 10.1016/0140-6736(93)92876-u

[93] Erdman JW Jr Balentine D Arab L Beecher G Dwyer JT Folts J Harnly J Hollman P Keen CL Mazza G Messina M Scalbert A Vita J Williamson G Burrowes J Flavonoids and heart health. In Proceedings of the ILSI North America Flavonoids Workshop, Washington, DC, USA, May 31–June 1, 2005 J. Nutr 2007 137 718S 737S 17311968 10.1093/jn/137.3.718S

[94] Buijsse B Feskens EJ Kok FJ Kromhout D Cocoa intake, blood pressure, and cardiovascular mortality: The Zutphen elderly study Arch. Intern. Med 2006 166 411 417 16505260 10.1001/archinte.166.4.411

[95] Mink PJ Scrafford CG Barraj LM Harnack L Hong CP Nettleton JA Jacobs DR Jr Flavonoid intake and cardiovascular disease mortality: A prospective study in postmenopausal women Am. J. Clin. Nutr 2007 85 895 909 17344514 10.1093/ajcn/85.3.895

[96] Hooper L Kroon PA Rimm EB Cohn JS Harvey I Le Cornu KA Ryder JJ Hall WL Cassidy A Flavonoids, flavonoid-rich foods, and cardiovascular risk: A meta-analysis of randomized controlled trials Am. J. Clin. Nutr 2008 88 38 50 18614722 10.1093/ajcn/88.1.38

[97] Janszky I Mukamal KJ Ljung R Ahnve S Ahlbom A Hallqvist J Chocolate consumption and mortality following a first acute myocardial infarction: The Stockholm heart epidemiology program J. Intern. Med 2009 266 248 257 19711504 10.1111/j.1365-2796.2009.02088.x

[98] Cooper KA Donovan JL Waterhouse AL Williamson G Cocoa and health: A decade of research Br. J. Nutr 2008 99 1 11 17666148 10.1017/S0007114507795296

[99] Mullen W Borges G Donovan JL Edwards CA Serafini M Lean ME Crozier A Milk decreases urinary excretion but not plasma pharmacokinetics of cocoa flavan-3-ol metabolites in humans Am. J. Clin. Nutr 2009 89 1784 1791 19403635 10.3945/ajcn.2008.27339

[100] Hollenberg NK Fisher ND Is it the dark in dark chocolate? Circulation 2007 116 2360 2362 18025400 10.1161/CIRCULATIONAHA.107.738070

